# Increased prevalence of transient visual disturbances with normal ocular exam findings in pregnancy – cross-sectional study

**DOI:** 10.1038/s41598-025-03279-y

**Published:** 2025-05-26

**Authors:** Noa Kapelushnik, Malachy Nemet, Maor Benjamin, Guy J. Ben-Simon, Daphna Landau Prat

**Affiliations:** 1 Orbital Ophthalmic Plastic & Lacrimal Surgery Institute, Goldschleger Eye Institute, Sheba Medical Center, Tel Hashomer, 52621 Israel; 2 Faculty of Medicine, Tel Aviv University, Ramat Aviv, Israel; 3 Sheba Talpiot Medical Leadership Program, Sheba Medical Center, Tel Hashomer, Israel

**Keywords:** Transient visual disturbances, Pregnancy, Eclampsia, Preeclampsia, Health care, Physical examination, Eye manifestations, Reproductive signs and symptoms

## Abstract

Pregnancy induces significant physiological changes in women, impacting various bodily systems. These alterations can influence ocular health and visual function. Ophthalmologists at our center noted an apparent increase in reports of transient binocular visual disturbances among pregnant women presenting to the Ophthalmic Emergency Room (OER), with no ophthalmic findings. This study aims to examine the rate of Transient Visual Disturbances with Normal Ocular Examination (TraViNo) in pregnant vs. non-pregnant women of childbearing age. A cross-sectional study was conducted at Sheba Medical Center, analyzing data from 562 females between 18 and 50 years of age who presented to the OER during January-March 2019. Data retrieved from the participants’ medical records included demographics, medical history, symptoms, comprehensive ophthalmological evaluation findings, and final diagnoses recorded at the OER. Included were 562 females (mean age 31.2 ± 9.0 years), among whom 69 (12.0%) were pregnant (mean pregnancy week 25 ± 11). The mean age was similar for both groups (*P* = 0.2). Presenting symptoms varied between the groups (*P* < 0.001), with the most common being visual disturbances in the pregnant group (PG) (58%). TraViNo was the final diagnosis in 86 (15%) study women and significantly more common in the PG (48% vs. 11%, respectively, *P* < 0.001). Most cases (74%) were bilateral. The additional workup included neurologic examinations in 45 (66%) cases, brain/orbital imaging in 28 (33%), and visual field testing in 8 (12%). Obstetric evaluations performed in the PG were normal except for 2 cases of hypertension. TraViNo is more common during pregnancy. Further research is warranted to identify underlying causes and long-term implications.

## Background

Pregnancy is a unique physiological state in which women undergo many physiological, emotional, and anatomical changes that affect almost every system in the body, including the visual system^1^.

Ophthalmologists in our ophthalmic emergency room (OER) observed a notable trend: pregnant women more frequently reported transient visual disturbances compared to men and non-pregnant women of childbearing age. Comprehensive ophthalmic evaluations, including visual acuity assessment, slit-lamp examination, funduscopic assessment, and, in some cases, supplementary examinations, such as automated perimetry, optical coherence tomography (OCT), and magnetic resonance imaging (MRI), failed to reveal any abnormal findings that could explain the visual symptoms. No structural or functional abnormalities were identified. In order to describe this phenomenon, we introduce the term “transient binocular visual disturbance with no ophthalmic findings” (TraViNo), defined as a temporary visual disturbance, with the absence of ophthalmic functional or structural abnormalities. Understanding TraViNo is clinically significant for multiple reasons. First, unexplained visual disturbances, even if transient, can cause considerable anxiety and distress, particularly due to concerns about underlying neurological or vascular conditions. Recognizing the benign nature of TraViNo, if confirmed, could reduce anxiety and unnecessary medical investigations and referrals. Lastly, these transient symptoms may reflect underlying systemic changes, warranting further exploration of pregnancy-associated vascular and neurological adaptations.

Pregnancy induces profound physiological changes. The hematological changes include rise in plasma volume, fall in hemoglobin counts (hemodilution) and in thrombocyte counts, and hypercoagulability^2^. Changes in the cardiovascular system manifest as cardiac output increases, the stroke volume increases, and higher heart rates are observed^3,4^. Endocrine changes include alterations in the function of the thyroid^5^, adrenal gland^6^, glucose metabolism^7^, and pituitary gland^8^. Vascular changes also occur during pregnancy, and they include proliferation, distention and instability of blood vessels^9^.These changes during pregnancy are primarily physiological, although they can also be associated with gestational diabetes, eclampsia, pre-eclampsia, stroke^10^, retinal emboli^11^, hypertension and arterial dissection^12^. All of these pathologies can have implications for the health of the eyes and affect the visual system^13^.

Visual changes during pregnancy are reported in the literature, some of which are related to hormonal changes, such as changes in cornea thickness^14^, decreased tear production^15^ and accommodation loss^16^. In addition, some changes can be a sign of pregnancy complications, such as pre-eclampsia^17^ or diabetes mellitus^18^. To the best of our knowledge, there are no studies on transient visual disturbances experienced by women during their pregnancy. This study aims to determine whether TraViNo is more prevalent during pregnancy and to explore possible systemic contributors to this phenomenon. By systematically investigating TraViNo, we hope to provide better guidance for clinicians evaluating pregnant women with transient visual symptoms, offering reassurance to patients while identifying any potential need for further assessment or intervention.

## Methods

### Patient population

This is a cross-sectional single center study of women admitted to the Sheba Medical Center OER during January-March 2019, thus obviating any bias caused by the Covid-19 pandemic^19^. Consecutive sampling was employed to reduce selection bias. all adult women of childbearing age (18–50 years old) who visited the OER between January-March 2019 were included. Data from each participant’s first OER visit during the study period were analyzed. Details on demographics, ocular and general medical history, the findings of a comprehensive ophthalmological evaluation and of supplementary assessments, and the final diagnosis at OER discharge were collected and evaluated.

Comprehensive ophthalmological evaluation included visual acuity by the Snellen chart, anterior and posterior segment evaluation by slit lamp biomicroscopy, intraocular pressure measured by a Goldmann tonometer, color vision assessment by the Hardy-Rand-Rittler test, pupil reaction assessment and extraocular movement assessment. The use of supplementary assessments, such as OCT, perimetric visual field, and MRI and CT scans were done according to the OER attending physician’s recommendations based upon each clinical case and not necessarily carried out in every case.

This research adhered to the tenets of the Declaration of Helsinki, and Institutional Review Board (IRB) of Sheba Medical Center approval was obtained (SMC-21-8400). The need for informed consent to participate was waived by Institutional Review Board of Sheba Medical Center.

### Statistical analysis

The Chi-square test was used to calculate proportional differences between categorical groups. An independent samples *t*-test was used to compare the means of independent continuous variables, such as age, visual acuity, and intraocular pressure. Multivariable regression analysis was used to adjust for cofounders, specifically logistic regression. In bilateral cases, only the right eye was included in the statistical analysis to avoid the influence of inter-eye correlations. Best-corrected visual acuity (BCVA) determined by means of the Snellen chart was converted to logarithm of the minimum angle of resolution (LogMAR) for the purposes of data analysis. The statistical analysis was carried out using SPSS (version 26, SPSS Inc., Chicago, IL). All results are presented as mean ± standard deviation (SD).

## Results

**Fig. 1:**
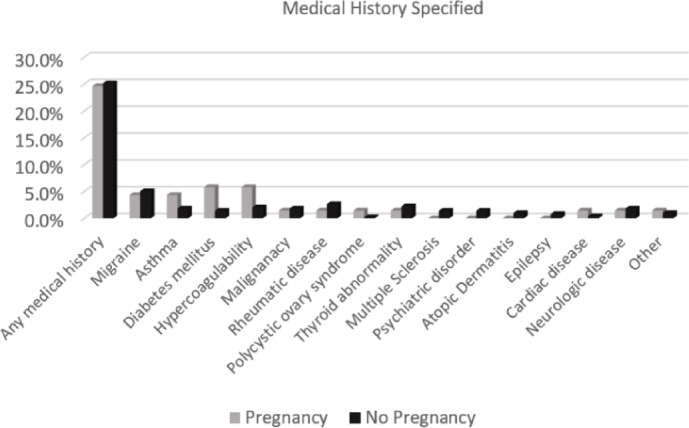
Diagnosis by pregnancy status.

The study cohort included a total of 562 females (mean age 31.2 ± 9.0 years, range 18–47 years). Of them, 324 (58%) had no ocular abnormalities, while 156 (28%) had refractive errors, most commonly myopia (151/156, 97%), and 28 (5%) of the latter had undergone refractive surgery. Other disorders which were documented in fewer than 3% of the study population included neuroophthalmological disorders (*n* = 12), corneal and ocular surface disorders including keratoconus (*n* = 4), dry eye (*n* = 3), resolved corneal abscess (*n* = 2), eyelid abnormalities including ptosis (*n* = 4), retinal abnormalities such as tear (*n* = 7) or detachment (*n* = 5), and 45 with other miscellaneous pathologies (e, g, uveitis, strabismus). Most (*n* = 421, 75%) of the women were healthy, and the others had a medical history of rheumatic diseases (*n* = 14, 3%), asthma (*n* = 12, 2%), hypercoagulability disorders (*n* = 14, 3%), neurologic disorders (*n* = 10, 2%), malignancy (*n* = 9, 2%), diabetes mellitus (*n* = 5, 1%), and miscellaneous disorders (e.g., cardiac pathologies, psychiatric disorders) (*n* = 74, 13%). Details of past medical history are outlined in Fig. 1.

### Pregnancy group (PG) and non-pregnancy group (NPG)

At the time of their OER visit, 69 (12.0%) women were pregnant (mean pregnancy week 25 ± 11, range 5–39 weeks). The mean age was similar for both study groups (*P* = 0.2, independent samples *t*-test). The pregnancy of 20 women (29%) was classified as ‘at risk’ due to various conditions, including multifetal pregnancy (*n* = 10) and pre-existing medical conditions such as thyroid disease (*n* = 1), rheumatic disease (*n* = 1), hypercoagulability (*n* = 4) and diabetes mellitus (*n* = 4). During the ER visit, one additional patient was diagnosed with preeclampsia, another with hypertension and another with pregnancy induced hypertension. Spectacle correction was used in about one-half of all cases in both groups (49% PG and 53% NPG, *P* = 0.7 χ^2^ test). A history of migraine headache (6% PG and 5% NPG) or other types of headaches (0% PG and 2% NPG) was similar between the groups (*P* = 0.5, χ^2^ test).

The presenting symptoms varied significantly between the 2 groups (*P* < 0.001, χ^2^ test), with the most common being visual disturbances in the PG (*n* = 40/69, 58%) compared to eye pain or foreign body sensation in the NPG (*n* = 248/493, 50%). symptoms were more often bilateral in the PG (*n* = 33/69, 48% PG vs. *n* = 137/493, 28% NPG, *P* < 0.001 χ^2^ test). Determination of the laterality of the symptoms was based upon the documented medical history provided by patients during their visits to the OER, which may be subject to misinterpretation and thus represents a potential source of diagnostic uncertainty. Visual disturbances were more common in the PG (*n* = 40/69, 58% vs. *n* = 147/493, 30% NPG, *P* = 0.005, χ^2^ test). Symptoms of flashes, floaters, and diplopia were similarly distributed between the groups (*P* > 0.05 for each, χ^2^ test), while symptoms of visual field disturbances were more common in the PG (*n* = 12/69, 17% vs. *n* = 43/493, 9% NPG, *P* = 0.04, χ^2^ test). Headache was more prevalent in the PG (*n* = 32/69, 46% vs. *n* = 80/493, 16% NPG, *P* < 0.001, χ^2^ test). Table 1 details the symptoms and ocular examination findings in both groups.

**Table 1:** Presenting symptoms, ocular examination findings, and final diagnoses in 562 females attending the ocular emergency room.

	Non-pregnant	Pregnant	Total	*P*-value*
N	493 (88%)	69 (12%)	562 (100%)	
Age (years)	30.4 ± 5.0	31.3 ± 9.5	31.2 ± 9.0	0.2**
Symptoms (N)				
Eye pain/foreign body sensation	248 (50%)	11 (16%)	259 (46%)	
Eyelid/orbital/lacrimal disorder	44 (9%)	2 (3%)	46 (8%)	
Visual disturbances	147 (30%)	40 (58%)	187 (33%)	
Asymptomatic - referred to ocular exam	43 (9%)	16 (23%)	59 (10%)	
Other	11 (2%)	0 (0%)	11 (2%)	
				Multivariate comparison < 0.001***
Symptoms – laterality(N)				
Right eye	179 (36%)	13 (19%)	192 (34%)	
Left eye	165 (33%)	13 (19%)	178 (32%)	
Both eyes	137 (28%)	33 (48%)	170 (30%)	
Asymptomatic/unknown	12 (2%)	10 (14%)	22 (4%)	
				Multivariate comparison < 0.001***
Symptom – associated details (N)				
Flashes	6 (9%)	18 (4%)	24 (4%)	0.1***
Floaters	6 (9%)	19 (4%)	25 (4%)	0.1***
Diplopia	1 (1%)	6 (1%)	7 (1%)	0.9***
Missing visual field	12 (17%)	43 (9%)	55 (10%)	0.04***
Headache	32 (46%)	80 (16%)	112 (20%)	< 0.001***
Ophthalmic exam				
Visual acuity LogMAR ± SD [Snellen]	0.04 ± 0.08 [20/22]	0.1 ± 0.3 [20/25]	0.1 ± 0.3 [20/25]	< 0.001**
IOP (mmHg)	13.1 ± 2.3	13.7 ± 3.0	13.5 ± 2.9	0.1**

Significant values are given in bold.

*Between the pregnancy and the non-pregnancy group.

**Independent samples *t*-test.

***χ^2^ test.

### Transient visual disturbances with normal ocular exam (TraViNo)

**Fig. 2:**
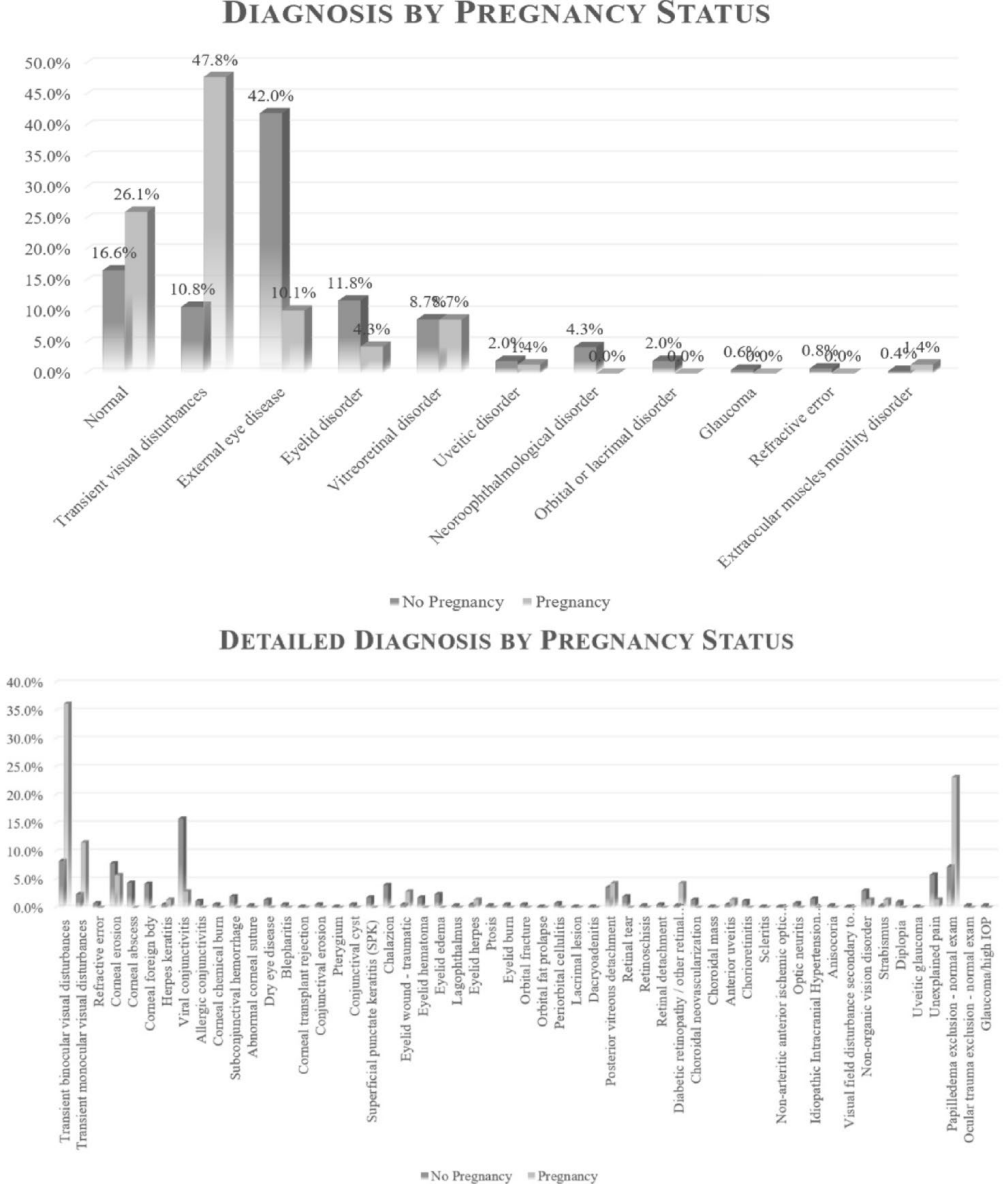
Final diagnoses of 561 female patients attending the ocular emergency room. (**A**) Diagnosis groups, (**B**) Detailed diagnoses. The diagnosis groups and detailed diagnosis rates differed between the group of pregnant women and the non-pregnant women (*P* < 0.001, χ^2^ test for both).

TraViNo was the final diagnosis in 86 (15%) of the study population, it was the most common diagnosis in the PG and less frequent in the NPG (*n* = 33/69, 48% vs. *n* = 53/493, 11% NPG, *P* < 0.001, χ^2^ test). To account for potential confounders such as medication use and medical history, a logistic regression analysis was performed. The association between pregnancy and TraViNo remained significant after adjustment, with an odds ratio of 14.37 (*p* < 0.001). A history of migraine headaches was documented in 16 (19%) of all cases and more common in the NPG group (*n* = 14/53, 26% vs. *n* = 2/33, 6% PG, *P* = 0.02, χ^2^ test). Figure 2 describes the final diagnosis of TraViNo, which varied between the 2 groups (*P* < 0.001, χ^2^ test). Most of the TraViNo cases were bilateral (*n* = 64/86, 74%). The women with TraViNo most commonly described non-specific visual disturbances or difficulty in focusing (as subjectively described by the patients. Scintillating scotoma was reported in 4 cases, zigzag lines in 3 cases, and geometric shapes in 2 cases. The visual disturbances were provoked in three cases: one by medication, one by prolonged exposure to a computer screen, and one by orthostatic changes. The most common visual disturbance was missing visual fields, recorded for 27 (31%) of the TraViNo cases. The duration of the visual disturbance was a matter of minutes in 45 (55%) cases, followed by hours in 11 (13%), days in 8 (10%), seconds in 6 (7%), and unspecified in the others.

Headache was associated with TraViNo in 48 (56%) cases. All the above-mentioned characteristics were similar for the 2 groups (*P* > 0.05 for all, χ^2^ test). Color vision and extraocular motility tests were normal in all cases, and no other acute ocular disorder was observed.

An additional neurologic examination during the first OER presentation was performed by a neurologist and/or ER physician in 45 (66%) cases, with no related abnormal findings. The cases who were examined by a neurologist and/or ER physician also had their blood pressure and body temperature measured. Brain/orbital imaging by computerized tomography or magnetic resonance imaging was performed in 28 (33%) cases (*n* = 4/33, 12% PG vs. *n* = 24/53, 45% NPG, *P* < 0.001 χ^2^ test). No further systemic evaluation, such as carotid US or electroencephalogram (EEG), was performed. A gynecologic exam was performed in 30 (35%) cases, 29 in the PG (*n* = 29/33, 87% PG vs. *n* = 1/53, 2% NPG, *P* < 0.001 χ^2^ test). All gynecologic findings were normal except for 2 cases in the PG group, one with a diagnosis of high blood pressure and one of pregnancy-induced hypertension. No cases of pre-eclampsia/eclampsia or hyperemesis gravidarum were documented. Visual field testing was performed in 8 (12%) cases: the findings were normal in 5 cases, unspecific changes were demonstrated in one case, mild unilateral upper arcuate defect in one case, and unreliable in one case. OCT of the optic nerve and macules was performed in 1 case and the result was normal. Table 2 provides further details and compares the characteristics of the TraViNo cases in both groups.

**Table 2:** Characteristics of 86 cases with transient visual disturbances with normal ocular exam (TraViNo) in both study groups.

	Pregnancy	Non-pregnancy	Total	*P*-value*
	33 (38%)	53 (62%)	86 (100%)	
	30.7 ± 5.2	33.0 ± 9.0	32.1 ± 7.8	0.1**
Right eye	6 (18%)	9 (17%)	15 (17%)	
Left eye	4 (12%)	3 (6%)	7 (8%)	
Both eyes	23 (70%)	41 (77%)	64 (74%)	
	2 (6%)	14 (26%)	16 (19%)	0.02***
History of migraine-associated symptoms (N)				
Flashes	5 (15%)	7 (13%)	12 (14%)	0.8***
Floaters	4 (12%)	2 (4%)	6 (7%)	0.3***
Diplopia	0 (0%)	1 (2%)	1 (1%)	1.0****
Missing visual field	8 (24%)	19 (36%)	27 (31%)	0.4***
Headache	19 (58%)	29 (55%)	48 (56%)	0.9***
Symptom duration				
Seconds	4 (12%)	2 (4%)	6 (7%)	0.1***
Minutes	18 (55%)	27 (51%)	45 (52%)	
Hours	4 (12%)	7 (13%)	11 (13%)	
Days	0 (0%)	8 (15%)	8 (9%)	
Unspecified	7 (21%)	9 (17%)	16 (19%)	
Additional exams				
Neurologic assessment	19 (58%)	26 (47%)	45 (66%)	0.2***
Gynecologic assessment	29 (87%)	1 (2%)	30 (35%)	< 0.001***
Brain/orbital imaging				
CT	1 (3%)	23 (43%)	24 (28%)	< 0.001***
MRI	3 (9%)	1 (2%)	4 (5%)	
None/unspecified	29 (88%)	29 (55%)	58 (67%)	
Visual field	1 (3%)	7 (20%)	8 (12%)	0.07***
OCT optic nerve and macules	0 (0%)	1 (3%)	1 (1%)	1.0***

Significant values are given in bold.

*Between the pregnancy and the non-pregnancy group.

**Independent samples t-test.

***χ^2^ test.

***Fisher’s-exact test.

## Discussion

Our impressions that ocular symptoms and diagnoses differ between pregnant and non-pregnant women of childbearing age who presented to the OER were borne out. The most common complaint among the pregnant women was visual disturbances, whereas the non-pregnant women predominantly reported ocular pain or foreign body sensation. Our findings indicate about one-half of pregnant women were diagnosed as having TraViNo compared to 11% of the non-pregnant women (*P* < 0.001). To the best of our knowledge, this is the first documentation of increased prevalence of TraViNo during pregnancy.

The effect of pregnancy on the ocular and visual system has been studied in depth. For example, women experiencing eclampsia or pre-eclampsia might sustain various levels of hypertensive retinopathy, ranging from minor alterations in the retinal blood vessels and the presence of cotton wool spots to more serious conditions, such as serous retinal detachment and papilledema^17^. Other examples of ocular conditions observed with a higher incidence during pregnancy are central serous retinopathy, which is probably due to the high cortisol levels during pregnancy^20^. Pathologies related to the orbit and eyelid also associated with pregnancy include ptosis^21^, Bell’s palsy^22^, enlargement of orbital hemangiomas, and carotid cavernous fistula formation^23,24^. However, none of those disorders commonly lead to TraViNo.

Some of the ocular and adnexal alternations during pregnancy are considered benign and do not require any treatment, since they mostly resolve during the post-partum period or after the cessation of breastfeeding^25,26^. They include changes in refraction due to corneal steepening and increase in corneal thickness^27–29^. Similarly, the crystalline lens is also affected during pregnancy due to an increase in the lens aquas component^30^ Moreover, the incidence of diabetes is higher among pregnant women in the form of gestational diabetes mellitus (GDM). Alternating blood glucose levels in women with GDM can cause transient blurred vision due to the osmotic effect on the crystalline lens^31^. Finally, a myopic shift of about one diopter can also occur during pregnancy^32^. Although these etiologies are associated with normal findings on the ocular examination, their impact persists throughout pregnancy, and are therefore incompatible with the transient nature of TraViNo, which lasted seconds to hours in most of the cases in this series.

The normal ocular examination findings recorded for the TraVino cases theoretically indicate the condition’s primary association with systemic conditions that lead to either temporary cortical or bilateral retinal or optic disc hypoperfusion. Such conditions can include migraine headaches, fluctuating blood glucose levels, vasovagal syncope, the Uhthoff phenomenon in demyelinating diseases, and posterior lobe epilepsy^33,34^. These phenomena cause various types of visual disturbances, ranging from difficulty in focusing due to fluctuating blood glucose levels and temporary osmotic changes that lead to refractive alterations, to tunnel vision in vasovagal episodes, scintillating scotoma in migraines, and others. Most of the women presented to our OER after their visual disturbances had subsided; subsequent neurological (*n* = 45, 66%), brain/orbital imaging (*n* = 28, 33%), and systemic examinations, albeit not routinely performed for all women presenting to the OER, yielded normal results. This theoretically narrows down the most likely contributing factors to bilateral TraViNo to migraine with visual aura, vasovagal syncope^35–37^ and, to a lesser extent, visual phenomena associated with epileptic seizures (although the latter appears less probable due to the absence of a reported post-ictal phase). These data suggest an association between pregnancy and a higher prevalence of TraViNo. Migraine with aura and vasovagal syncope may be contributing factors but require further investigation. These conditions, which can be influenced by pregnancy and are generally reversible, also warrant further investigation. It is important to stress that none of the women underwent additional systemic evaluations, such as carotid US or EEG, which could have provided further insights into potential vascular- or seizure-related causes of TraViNo. This limits the ability to rule out vascular and neurological contributors to TraViNo.

Migraine with aura is reportedly more common during pregnancy^38^. Robbins et al. found that the initial occurrence of migraine with aura for many women occurred during their pregnancy^39^. In the current study, although coexisting headache rates were similar between the group of pregnant women with TraViNo and the non-pregnant women with TraVino, headaches were much more prevalent in the former group (46% vs. 16%, respectively, *P* < 0.001). This supports the hypothesis that migraine or ocular migraine may be one of the primary mechanisms causing increased rates of TraViNo during pregnancy. Visual disturbances during migraine-related aura manifest in a wide range of symptoms, including flashes of bright light, blurred vision, zigzag lines, scotomas, black dots, small bright dots (phosphenes), flickering lights, tunnel vision, circular shapes, oscillopsia, and more^35^.

A vasovagal aberration may be another potential etiology for TraViNo. The numerous cardiovascular changes that take place during pregnancy predispose pregnant women to presyncope and syncope through an exaggerated vasovagal response mechanism^40,41^. As discussed by Fu et al., pregnancy is associated with profound and early changes in autonomic regulation and vascular tone including increased sympathetic outflow and decreased systemic vascular resistance, transient autonomic dysregulation may contribute to vasovagal response. The interplay between increased sympathetic drive and attenuated vascular responsiveness may lead to transient hemodynamic instability. Specifically, this autonomic imbalance—especially during early gestation when cardiovascular adaptations are still evolving—may predispose susceptible individuals to transient visual symptoms^42^. During those episodes, the low blood pressure may cause transient visual disturbances, which could manifest independently and not necessarily precede loss of consciousness. In pre-syncope, the visual disturbances are characterized by a shrinking of the peripheral visual field, evolving into tunnel vision and culminating in complete loss of sight^31^. The mechanism by which the vision is affected in vasovagal syncope is thought to be due to hypoperfusion to the retina when the cerebral perfusion is adequate. The presence of intraocular pressure restricts eye perfusion during blood hypotension, a factor that is absent in the brain^43^. It is worth noting that although the vasovagal response during pregnancy is generally considered benign^40^, syncope may be associated with pregnancy complications^41^. The ocular effects of TraViNo linked to syncope await further investigation.

Epilepsy can manifest as transient visual disturbances, including transient visual loss and visual hallucinations, which may be simple (e.g., flashes or colored dots) or complex (involving detailed images or objects). Misdiagnosis is a recognized concern due to the similarity of these symptoms to migraine-related visual aura^44^ None of the women diagnosed with TraViNo in this study underwent an evaluation to rule out seizure as a potential cause. This may be attributed to the absence of additional relevant features, such as a postictal phase, which could have raised suspicion for seizure activity.

Despite the reassuring results of this study that most of the pregnant women who complain of visual disturbances eventually had a normal ophthalmic exam, it remains mandatory to conduct thorough eye examinations for all female patients, both pregnant and non-pregnant ones who complain of visual disturbances. Serious ocular, obstetric, and systemic conditions can manifest visual disturbances, especially during pregnancy.

This study has several limitations. First, it is retrospective which inherently restricts the ability to apply a standardized and systematic diagnostic approach across all patients. In the absence of a unified protocol for evaluating transient visual disturbances with normal ocular examinations (TraViNo), the diagnostic tools and techniques that had been employed were based upon the clinical judgment of the attending physicians. As a result, not all patients underwent the same set of diagnostic tests or the use of advanced tools. Since this retrospective study relies upon medical records from OER visits, the information on past medical history is based upon the medical history provided by patients and the details recorded in their medical charts. This inherently limits the completeness and availability of data regarding medical history and conditions potentially linked to transient visual loss. Second, there is no long-term follow-up information for the women who were diagnosed in the OER as having TraViNo, thus limiting the assessment of long-term outcomes and additional workup results, which could provide further insight into the causality of TraViNo and its clinical implications. Theoretically, such workups could include advanced imaging, such as CT or MRI, EEG, as well as OCT and visual field tests which were available for only a minority of cases in this study. Third, data on visual field and OCT tests were available for only a minority of cases. Lastly, the medical center in which the research was conducted is one of the largest referral medical centers nationwide. Therefore, there might be a selection bias, with more complex cases presenting to our OER and the increased rates of referral to ocular exams from other departments, including gynecology. This could potentially bias the findings and limit the generalizability of the study’s conclusions to other, less specialized healthcare settings. Additionally, the more extensive availability of comprehensive ophthalmic evaluations in a tertiary care center may have influenced the observed prevalence of TraViNo, further skewing the results.

## Conclusion

Most of the pregnant women who presented to our OER for an ophthalmological exam were diagnosed as having TraViNo compared to non-pregnant women of child-bearing age. Potential causes for TraViNo are migraines with aura or vasovagal syncope. Other conditions with significant health implications, such as epileptic seizures, may also be contributing factors, and they require further validation. Implementing a standardized protocol for the systemic and ocular assessment of patients presenting with TraViNo could provide valuable insights into its underlying causes, suitable treatments, and appropriate follow-up. Further research is warranted to enhance our understanding of TraViNo and its potential long-term implications.

## Data Availability

The data that support the findings of this study are not openly available due to reasons of sensitivity and are available from the corresponding author upon reasonable request.

## Abbreviations

OER: Ophthalmic emergency room
TraViNo: Transient visual disturbances with normal ocular examination
PG: Pregnant group
NPG: Non pregnancy group
MRI: Magnetic resonance imaging
OCT: Ocular coherence tomography
EEG: Electroencephalography
BCVA: Best-corrected visual acuity
LogMAR: Logarithm of the minimum angle of resolution
GDM: Gestational diabetes mellitus
